# The Effect of Corneal Refractive Power Area Changes on Myopia Progression during Orthokeratology

**DOI:** 10.1155/2022/5530162

**Published:** 2022-06-16

**Authors:** Minfeng Chen, Xinting Liu, Zhu Xie, Pengqi Wang, Miaoran Zheng, Xinjie Mao

**Affiliations:** School of Ophthalmology and Optometry, Wenzhou Medical University, Wenzhou, Zhejiang 325000, China

## Abstract

**Purpose:**

To investigate the effect of corneal refractive power area changes on myopia progression during orthokeratology.

**Methods:**

One hundred and sixteen children who met the inclusion criteria and insisted on wearing orthokeratology lenses for two years were retrospectively assessed. Seventy-two children with the orthokeratology lens decentration distance more than 0.5 mm but less than 1.5 mm were in the decentered group, and forty-four children with the orthokeratology lens decentration distance less than 0.5 mm were in the centric group. The orthokeratology decentration via tangential difference topography was analyzed. This study calculated the different power areas in the central 4 mm pupillary area by axial-difference corneal topography, compared the differences of the different power areas between these two groups, and evaluated the relationships between corneal positive-power area, orthokeratology decentration, and AL changes.

**Results:**

The axial length changes of the centric group presented a statistical difference with the decentered group (0.52 ± 0.37 mm vs. 0.38 ± 0.26 mm; *t* = 2.403, *p*=0.018). For all children, both the AL changes (0.43 ± 0.31 mm) and decentration distance (0.64 ± 0.33 mm) showed a significant correlation with the positive-power area (*r* = −0.366, *p* < 0.001 and *r* = 0.624, *p* < 0.001); AL changes also presented a statistical correlation with decentration distance (*r* = −0.343, *p* < 0.001), baseline age (*r* = −0.329, *p* < 0.001), and baseline spherical equivalent refractive power (*r* = 0.335, *p* < 0.001). In the centric group and decentered group, the AL changes (centric group: *r* = −0.319, *p*=0.035; decentered group: *r* = −0.332, *p*=0.04) and decentration distance (centric group: *r* = 0.462, *p*=0.002; decentered group: *r* = 0.524, *p* < 0.001) had a significant correlation with the positive-power area yet. In the multiple regression analysis, AL changes were increased with less baseline age (beta, 0.015; *p* < 0.001), positive-power area (beta, 0.021; *p*=0.002), and larger SER (beta, 0.025; *p*=0.018).

**Conclusions:**

The corneal positive-power area had a positive impact on affirming AL changes during orthokeratology. This area might be formed by lens decentration to provide an additional myopia-defocusing influence on the retina to achieve better myopia control.

## 1. Introduction

Currently, there are around 1.4 billion myopic people in the world, nearly half of whom live in China. Without effective intervention, it is estimated that by 2050, there will be 4.758 billion children with myopia, accounting for about half of the total population [1, 2]. Thus, myopia is a global public health concern. Many studies have confirmed that orthokeratology is a safe, effective, and reversible method for slowing axial length (AL) changes [3–5]. After orthokeratology treatment, AL changes are reduced by 30–50% [3–6], effectively mitigating the development of myopia.

Orthokeratology changes the corneal surface by flattening the central cornea and steepening the mid-peripheral cornea to provide a clear unaided distance vision and myopia control; this is achieved through the application of gas-permeable contact lenses [6–8]. However, the mechanism underlying the effect of orthokeratology on the control of myopia progression remains unknown. Moreover, the effects of orthokeratology on mitigating AL changes vary significantly among children. Many factors [8–10] influence the level of myopia control achieved with orthokeratology, including baseline age, baseline spherical equivalent refractive power (SER), and pupil diameter. Zhong et al. also found that changes in corneal refractive power significantly affected AL changes; specifically, subjects with a larger magnitude of corneal refractive power change after orthokeratology treatment typically experienced slower AL changes [11–16].

Following orthokeratology treatment, the cornea becomes a multifocal cornea [6]. Hiraoka et al. found that corneal multifocality was negatively correlated with the AL change, whereas patients with increased corneal multifocality showed less AL elongation [13–17]. Hiraoka et al. suggested that corneal multifocality might be affected by lens decentration. Recent studies [11, 12] have also revealed that lens decentration affects myopia control during orthokeratology. Wang and Yang [11] found that acceptable lens decentration could delay the development of myopia more effectively than when the lens is in a centric position.

In previous studies [11–20], both corneal refractive power change and lens decentration have been shown to have an effect on AL changes, and lens decentration may have an effect on changes in the corneal refractive power [11, 12, 20, 21]. Thus, it is worth investigating whether there might be relationships between decentration, corneal refractive power changes, and AL changes.

To this end, the current study calculated lens decentration using tangential corneal topography and analyzed the areas of corneal refractive power change during orthokeratology using axial corneal topography [18–23]. Then, the relationships between lens decentration, AL changes, and corneal refractive power changes were investigated.

## 2. Methods

### 2.1. Study Subjects

A retrospective analysis was performed on 116 children (66 males and 50 females), ranging in age from 8 to 14 years (mean age, 11.19 ± 1.63 years). The subjects were all treated at Wenzhou Medical University Eye Hospital between June 2016 and October 2018 and were asked to wear orthokeratology lenses for no less than 8 hours per night over a two-year period. The inclusion criteria were as follows: age 8–15 years; spherical refractive power (SR) of −1.00 to −6.00 diopters (D); astigmatism of 0.00 to −1.50 D; binocular best-corrected visual acuity of 20/20 or greater; intraocular pressure of 10 to 21 mmHg; lens-fitting decentration less than 1.5 mm, to prevent the sclera from being covered [6, 11]; no other eye diseases; no history of surgery or use of atropine or contact lenses to control myopia progression; no systemic or ocular conditions that might affect vision. This study was performed in accordance with the tenets of the Declaration of Helsinki and was approved by the Ethics Committee of Wenzhou Medical University Eye Hospital.

### 2.2. Instrumentation

The orthokeratology lenses used in this study were four-zoned reverse-geometry lenses (Euclid Systems Corporation, USA). These lenses range in diameter from 10.2 mm to 11.2 mm and have a central thickness ranging from 0.22 mm to 0.23 mm. Each subject underwent a comprehensive baseline eye examination, including slit-lamp examination, refraction, uncorrected visual acuity, best-corrected visual acuity, AL (ZEISS IOLMaster; Carl Zeiss AG, Oberkochen, Germany), corneal topography (E-300; Medmont International Pty. Ltd.), corneal endothelium microscopy, and intraocular pressure (NT-2000; NIDEK CO., LTD., Gamagori, Japan).

All children were treated by doctors who had worked in the field of orthokeratology treatment at the eye hospital for more than 10 years. Corneal topography was measured with a Medmont E300 (Medmont International Pty. Ltd.); measurement was performed by a specialized technician within one hour of removal of the orthokeratology lenses. The topographic images used for analysis were each subject's best focus image (with an accuracy greater than 95%) from the four frames that were captured automatically. Based on each subject's corneal topography and the fitting evaluation based on corneal fluorescein pattern analysis, the doctor ordered lenses for the subject that were the most suitable based on their corneal parameters, according to the manufacturer's guidelines.

### 2.3. Measurements

Through corneal topography, the areas of different refractive powers within the central 4 mm pupillary area were calculated and recorded as the refractive distribution of the cornea; this is used as an index of corneal multifocality [6, 13, 20–24]. In all subjects, the right eye was selected for analysis.

**Figure 1:**
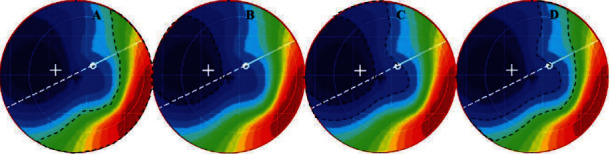
Areas of different refractive powers in the central 4 mm pupillary area ((a) positive-power area, (b) overcorrected area, (c) corrected area, and (d) undercorrected area).

Based on the recorded power areas of the pupillary area obtained from axial difference topography, the following measurements were made: positive-power area (positive D value), corrected area (D value = baseline SER ± 0.25 D), overcorrected area (D value > baseline SER − 0.25 D), and undercorrected area (D value < baseline SER + 0.25 D). To calculate the different refractive powers in the central 4 mm pupillary area, axial difference topography was performed, based on the axial topography [25], at the baseline and after three months of continuous orthokeratology lens wear. According to the axial difference topography, the refractive power parameter was set as the individual baseline SER power (Figure 1).

To assess the optical zone decentration distance, the tangential difference topography was constructed through tangential topography measurements collected at the baseline and after three months of continuous orthokeratology. According to the tangential difference topography, the optical zones ranged from the corneal vertex to (1) where the keratometry values changed within 1 D and (2) there were less than two colors (red and blue) in the refractive power parameter [18–21].

**Figure 2:**
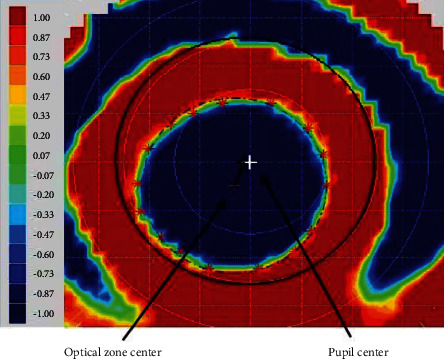
Optical zone decentration distance measurement (decentration distance was measured from the center of the optical zone to the pupil center).

After importing the tangential difference topography into MATLAB (MathWorks, Inc), 16 points were selected in a clockwise fashion, on the red-blue transition zone, to delineate the margins of the optical zone [18, 26, 27]. The pupil center was determined by the Medmont E300. The decentration distance of orthokeratology was defined from the center of the optical zone to the pupil center [12, 13, 28, 29] and was measured using ImageJ software (National Institutes of Health, Bethesda, MD, USA) (Figure 2). Based on the decentration distance, participants were divided into a centric group (decentration distance less than 0.5 mm) and decentered group (decentration distance larger than 0.5 mm).

### 2.4. Statistical Analysis

The degree of myopia was expressed by the SER. Measurement data are expressed as mean ± standard deviation (SD). Intragroup comparisons were performed using the paired *t*-test and intergroup comparisons were performed using analysis of variance or the independent *t*-test; the Wilcoxon rank-sum test for two related samples was used to compare data across time points. Pearson correlation analysis was performed to examine the relationships between the variables. A *p* value less than 0.05 was considered a statistically significant difference. Statistical Package for the Social Sciences (SPSS) version 25 software (IBM Corporation, Armonk, NY, USA) was used for the statistical analyses.

## 3. Results

### 3.1. Study Subjects

A total of 116 myopic children (mean age, 11.19 ± 1.63 years, range 8 to 14 years) were included in the sample. Before orthokeratology, the SER, SR, and regular astigmatism values were −3.56 ± 1.21 D (range −6.00 to −1.00 D), −3.41 ± 1.17 D (range −6.00 to −1.00 DS), and −0.36 ± −0.40 D (range −1.50 to 0.00 DC), respectively. The baseline parameters for both the centric group (decentration distance: 0.31 ± 0.13) and decentered group (decentration distance: 0.84 ± 0.25) are shown in Table 1.

**Figure 3:**
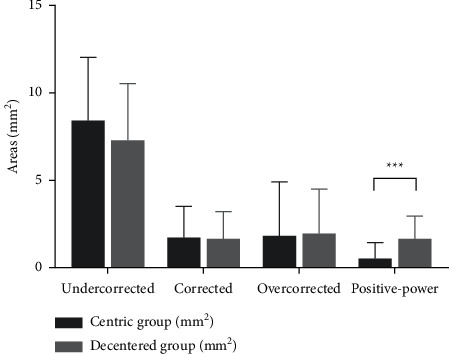
Comparison of the distribution of different refractive power areas between the centric group and decentered group (^*∗∗∗*^*p* < 0.001).

Among the total sample, significant differences were observed between the baseline and two-year AL change (25.07 ± 0.81 mm vs. 25.50 ± 0.76 mm; *t* = −12.547, *p* < 0.001). The AL change in the centric group was significantly different from that of the decentered group (0.52 ± 0.37 mm vs. 0.38 ± 0.26 mm; *t* = 2.403, *p*=0.018). After orthokeratology, the distributions of the different refractive power areas in the pupillary areas of these two groups are shown in Figure 2. There were no statistically significant differences in the following parameters between the centric group and decentered group: undercorrected area (8.49 ± 3.56 mm^2^ vs. 7.37 ± 3.20 mm^2^, *t* = 1.745, *p*=0.084), corrected area (1.72 ± 1.71 mm^2^ vs. 1.66 ± 1.50 mm^2^, *t* = 0.190, *p*=0.850), and overcorrected area (1.79 ± 3.01 mm^2^ vs. 1.90 ± 2.49 mm^2^, *t* = −0.215, *p*=0.830). However, the positive-power area (0.51 ± 0.86 mm^2^ vs. 1.61 ± 1.27 mm^2^, *t* = −5.087, *p* < 0.001) significantly differed between the centric group and the decentered group, as shown in Figure 3.

**Figure 4:**
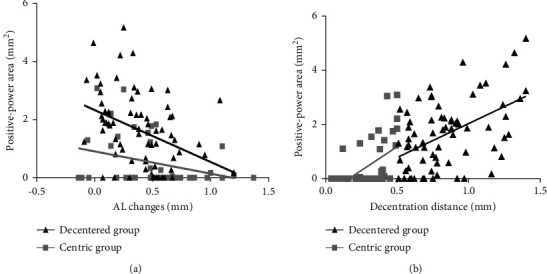
Correlation analysis of AL change and decentration distance with the positive-power area in the centric group and decentered group ((a) correlation between AL change and positive-power area in the centric group and decentered group; (b) correlation between decentered distance and positive-power area in the centric group and decentered group).

Among the 116 children, both the AL change (0.43 ± 0.31 mm) and decentration distance (0.64 ± 0.33 mm) were significantly correlated with the positive-power area (*r* = −0.366, *p* < 0.001 and *r* = 0.624, *p* < 0.001, respectively). Further, AL change was significantly correlated with the decentration distance (*r* = −0.343, *p* < 0.001), baseline age (*r* = −0.329, *p* < 0.001), and baseline SER (*r* = 0.335, *p* < 0.001). In the centric group and decentered group, the AL change (centric group: *r* = −0.319, *p*=0.035; decentered group: *r* = −0.332, *p*=0.04) and decentration distance (centric group: *r* = 0.462, *p*=0.002; decentered group: *r* = 0.524, *p* < 0.001) were significantly correlated with the positive-power area, as shown in Figure 4.

The relationships between baseline age, baseline SER, decentration distance, different power areas, and AL change among the total sample of 116 children are shown in Table 2. In the multiple regression analysis, AL change was associated with younger baseline age (beta, 0.015; *p* < 0.001), positive-power area (beta, 0.021; *p*=0.002), and larger SER (beta, 0.025; *p*=0.018). However, the decentration distance, overcorrected area, and corrected area were not significantly associated with AL change in the multiple linear regression analysis, as shown in Table 2.

## 4. Discussion

Orthokeratology is a safe and effective method for slowing changes in the AL [3–5], and more and more children and parents are choosing orthokeratology to prevent increases in myopia. However, the effect of orthokeratology on myopia control is influenced by eyelid tension, lens movement, and corneal asymmetry, among other factors. As a result, decentration [11, 12, 20, 21] has become a common phenomenon and cannot be completely avoided during orthokeratology treatment. Recently, studies [11, 12] have found that decentration might also have an effect on myopia control, in addition to the above previously known factors.

In the study by Wang and Yang [11], after orthokeratology, the AL change in the lens decentered group was smaller than that in the lens centric group. Moreover, Chen et al. [12] found a negative correlation between the lens decentration distance and AL change. In the current study, the myopic children wore orthokeratology lenses for two years and did not experience any serious complications that required them to stop wearing the lenses. In the sample of the present study, the mean decentration distance was 0.64 ± 0.33 mm (range: 0.38–1.40 mm), similar to the distances of 0.73 ± 0.25 mm (range: 0.5–1.50 mm) and 0.64 ± 0.38 mm (range: 0.13–1.78 mm) reported in the studies conducted by Wang and Yang [11], and Chen et al. [12]. Furthermore, consistent with other recent studies [11, 12], in the current study, in addition to the correlations between AL change and both baseline age and baseline SER, children with a greater decentration distance exhibited less change in AL after orthokeratology.

Modern orthokeratology is a process that uses reverse-geometry-designed rigid contact lenses to reshape the eye over time. Each lens had a back optical zone diameter of 6.0 mm, a reverse curve of 0.6 mm in width, an alignment curve of 1.25 mm in width, and a peripheral curve of 0.4 mm in width [14, 25, 28]. After the base curve flattens the cornea, ideally, a flat zone is formed to correct the refractive error. Unfortunately, after orthokeratology, the cornea becomes a multifocal cornea with many different refractive power areas in this zone, including an overcorrected area, corrected area, and undercorrected area. Hiraoka et al. [14] showed that greater decentration caused a greater change in corneal multifocality.

According to previous studies, the 4 mm area in the center of the pupil is the effective applanation zone. To investigate the relationships between the decentration distance and AL change, the areas of different refractive power in the central 4 mm pupil area were investigated using corneal topography. The results indicated that, compared with the centric group, the decentered group had a greater positive-power area in the central 4 mm pupil area, and the positive-power area was significantly negatively correlated with the decentration distance in both the centric group and decentered group. However, there were no significant differences in the undercorrected area, corrected area, and overcorrected area between the centric group and decentered group. The reversal curve, which is next to the base curve, forms the positive-power area in the cornea; thus, when the position of the orthokeratology lens is decentered, there is a greater positive-power area in the central 4 mm pupil area.

Correlation analysis indicated that the positive-power area was negatively correlated with the AL change, with a greater positive-power area associated with less AL elongation. This is consistent with the relationship between the decentration distance and AL change reported in previous studies [11, 12], where a larger decentration distance was associated with a greater positive-power area. In the univariate linear regression analysis, the decentration distance and positive-power area were significantly associated with AL change, but in the multiple linear regression analysis, the positive-power area, and not the decentration distance, was significantly associated with AL change. Thus, it appears that decentration changes the corneal refractive power of the pupil area, impacting the positive-power area and, in turn, influencing AL change.

Lam et al. [29] found that a custom-made spectacle lens (composed of a central optical zone for correcting the distance refractive error and an annular multifocal zone with multiple segments having a relative positive power) could effectively slow myopia progression. Lam et al.'s study confirmed that paracentral myopia defocus is effective for myopia control. Today, children have to work at a close range to their viewing target for long durations, due to the increasing burden of schoolwork. In this context, although myopia may be corrected with orthokeratology, these children may still experience a certain degree of adjustment lag [30–32], which might cause the fundus image to form behind the retina instead of on the retina, leading to hyperopia defocus [33, 34] on the retina; this would, in turn, accelerate the progression of myopia. However, among children with positive-power areas in the pupil zone, it appears that there is a positive-power lens in front of the cornea, providing paracentral myopia defocus on the retina; this would move the image from behind the retina to onto the retina or even in front of the retina, turning the hyperopia defocus into myopia defocus [33, 34]. This would result in an additional degree of myopia defocus on the paracentral retina, and thus, would provide a better myopia-control effect.

One limitation of the current study is that the measurement of lens decentration was only performed after the treatment had begun. Although previous studies have confirmed that there are no significant changes in decentration after two years of treatment, it would be valuable to know the magnitudes of decentration and positive-power area changes during the course of orthokeratology. Another limitation is that the degree of defocus could not be measured according to the positive-power area. This makes it difficult to decide how large the positive-power area should be in order to provide the best myopia control treatment effect. Additional studies should be performed to clarify these points.

## 5. Conclusions

In summary, after orthokeratology lens wear, optical zone decentration is common and unavoidable. A greater decentration distance might change the corneal refractive power of the pupil area, resulting in a certain degree of positive-power area. This might, in turn, change hyperopia defocus into myopia defocus, resulting in an additional degree of myopia defocus on the paracentral retina. This would be helpful for children when performing their schoolwork at a close range, thus providing a better myopia-control effect.

## Figures and Tables

**Table 1:** The baseline parameters between the centric group and decentered group (mean ± SD).

Groups	Centric position (*n* = 44)	Decentered position (*n* = 72)	*t*	*p*
Age (*y*)	10.86 ± 1.84	11.39 ± 1.47	−1.697	0.092
SR (DS)	−3.27 ± 1.22	−3.49 ± 1.15	0.967	0.336
Astigmatism (DC)	0.37 ± 0.43	0.36 ± 0.39	−0.151	0.880
SER (D)	−3.41 ± 1.31	−3.64 ± 1.14	0.985	0.327
AL (mm)	25.14 ± 0.83	25.03 ± 0.81	0.573	0.569
Flat-K (D)	43.02 ± 1.24	42.63 ± 1.24	1.546	0.125
Steep-K (D)	44.22 ± 1.23	44.08 ± 2.07	0.378	0.706
ΔK (D)	1.20 ± 0.44	1.30 ± 0.63	−0.847	0.399

*Note*. SR: spherical refractive power; SER: spherical equivalent refractive power; AL: axial length; flat-K: flat radial refractive power; steep-K: steep radial refractive power; ΔK: the difference between flat-K and steep-K.

**Table 2:** Univariate and multiple linear regression analysis to determine the correlations between AL change and baseline age, baseline SER, decentration distance, and the different power areas.

	Univariate linear regression			Multiple linear regression		
	B	*β*	*p*	B	*β*	*p*
Age (y)	−0.063	−0.329	<0.001	−0.055	0.015	<0.001
SER (D)	0.086	0.335	<0.001	0.066	0.021	0.002
Decentration distance (mm)	−0.325	−0.343	<0.001			
Overcorrected area (mm^2^)	0.022	0.194	0.037			
Corrected area (mm^2^)	0.046	0.234	0.011			
Positive-power area (mm^2^)	−0.091	−0.366	<0.001	−0.060	0.025	0.018

*Note*. AL: axial length; SER: spherical equivalent refractive power.

## Data Availability

The data used to support the findings of this study are available from the corresponding author (Xinjie Mao) upon request.
